# Blockchain-powered grids: Paving the way for a sustainable and efficient future

**DOI:** 10.1016/j.heliyon.2024.e31592

**Published:** 2024-05-21

**Authors:** Nazir Ullah, Waleed Mugahed Al-Rahmi, Fahad Alblehai, Yudi Fernando, Zahyah H. Alharbi, Rinat Zhanbayev, Ahmad Samed Al-Adwan, Mohammed Habes

**Affiliations:** a School of Management and Engineering, Nanjing University, Nanjing, 210093, China; b Faculty of Social Science and Humanities, School of Education, University Teknologi Malaysia, Skudai, 81310, Johor, Malaysia; c Computer Science Department, Community College, King Saud University, Riyadh, 11437, Saudi Arabia; d Faculty of Industrial Management, Universiti Malaysia Pahang Al-Sultan Abdullah, 26300, Malaysia; e Management Department, BINUS Online Learning, Bina Nusantara University, 11530, Indonesia; f Department of Management Information Systems, College of Business Administration, King Saud University, Riyadh, 12372, Saudi Arabia; g National Engineering Academy of the Republic of Kazakhstan, Almaty, Kazakhstan; h Department of Business Technology, Hourani Center for Applied Scientific Research, Business School, Al Ahliyya Amman University, Amman, Jordan; i Faculty of Mass Communication, Radio & TV Department, Yarmouk University, Irbid, P.O., 21163, Jordan

**Keywords:** Blockchain, TOE, Distributed ledgers, Energy, Smart contracts

## Abstract

Blockchain technology can potentially be a bedrock for the energy record-keeping system. This study examined several critical factors that affect users’ intention to accept Blockchain technology for the Smart grid. The proposed model is based on the Technology Organization Environment framework, which is examined using Structural Equation Modelling. Based on the study findings, it has been indicated that the relative advantage shows a significant effect and matters the most during the Blockchain technology adoption in the Smart grid. The innovativeness, cost saving, and regulatory support also significantly influence the intention to use the Blockchain technology. The innovativeness and traceability show a significant influence on upper management support. The traceability shows a substantial impact on cost savings. However, innovativeness shows an insignificant effect on cost savings. The traceability and competitive pressure do not affect the intention to use the Blockchain technology. This study has extended the Technology Organization Environment theory, which predicts the organizational behavior to adopt the Blockchain technology for the Smart grid. We argue that the finding provides insights to guide the industry to deliver the best practice on the Blockchain technology. The study findings suggest that experts would recognize innovative technology free of effort to raise the determined aids for improving the traditional energy system. Though there are some limits, theoretical and practical implications are justified based on the findings.

## 1 Introduction

Any country's economic success and achievement on environmental sustainability depend on digitalization and technological advancement. The industry and government sector around the globe are adopting current methods and digitalization technologies to strengthen the competitiveness to sustain strategically [1]. The energy sector is critical for achieving efficiency and meeting the country's and its citizens' demands. Between 2015 and 2040, global energy consumption and requirements are expected to rise by 28 percent. The predicted increase in energy in the Asian region is 51 percent, the largest among all other areas in the world. Energy generation and distribution are currently causing severe challenges in emerging countries. Meeting the energy needs of such countries' industrial and residential sectors is presently a significant concern. The energy crisis in Pakistan has affected 25 million people, and it is currently a significant challenge to meet the energy needs of the manufacturing and domestic areas [2].

**Fig. 1:**
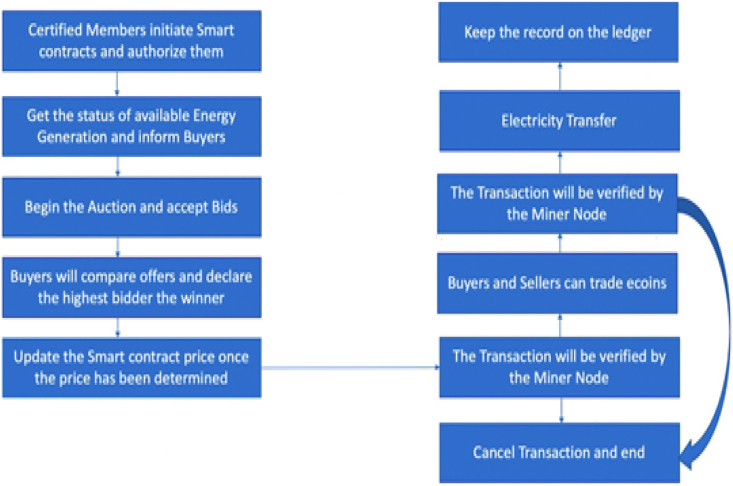
Peer-to-Peer Energy trading [4].

The effect of energy generation on the environment has been systematically studied to promote novel technologies that use renewable energy sources rather than fossil fuels. Integrating intermittent power from wind or solar plants into the energy distribution system is complicated. Because of the dispersed nature of those physical assets, the scholars need to rethink to improve the effectiveness of current energy management system. Blockchain technology is a viable way to provide workable solutions. The implementation of Blockchain technology in energy trade will have a significant positive impact on energy sustainability by delivering increased customer convenience. The autonomous peer-to-peer energy trading process via blockchain applications is worth mentioning. On the other hand, real-time energy trading applications necessitate safety and speed. The existing energy transaction method has little data privacy because the recorded transaction information is altered if the dominant authority is concerned. At the same time, blockchain data is maintained through each node in the system network. Furthermore, dynamically participating in grid operations from multiple locations is challenging with a centralized server due to recordkeeping and verification checks. Again, relying on a sole central energy provider can limit scalability. According to K. Shuaib et al. [3], integrating Blockchain technology for energy exchange can overcome scalability and flexibility issues. Prosumers can exchange nearby produced energy from renewable resources to buyers or a microgrid in a decentralized energy trading market. A billing model is another blockchain-based technique that could be used for the peer-to-peer energy exchange. A trustworthy billing mechanism can be adopted in public areas so anyone can use it. A decentralized energy transaction network can avoid electricity loss due to long transmissions. Energy transactions have economic aid since they make the energy market more competitive for energy manufacturers. Without depending on intermediaries, Blockchain combined with smart contracts could permit an autonomous and transparent way of trading energy directly to customers. A smart contract could facilitate safe and automated energy transactions by conducting bidding and payment processes. Consumers can look for low-cost distributors using a transparent methodology. Energy exchanges are recorded on distributed ledgers at any given time. Smart contract payments can automatically do energy trading without the need for human intervention. In general, Fig. 1 illustrates the basic procedure of the peer-to-peer energy system by utilizing smart contracts.

**Fig. 2:**
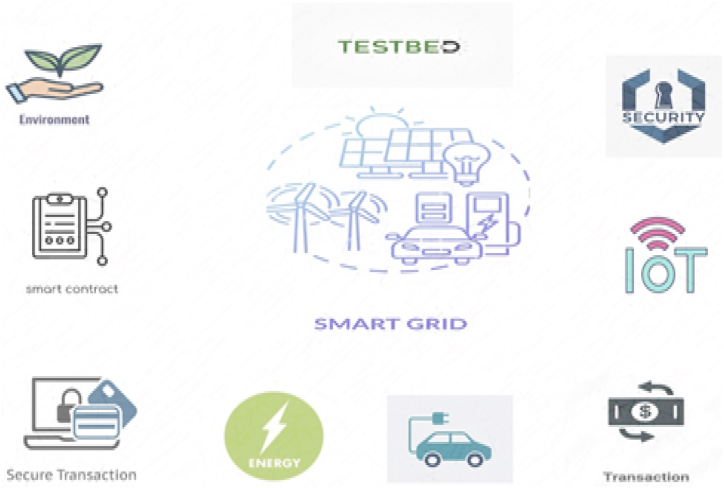
Blockchain applications in smart grid

The Smart grid's unique characteristics, such as the assimilation of renewable energy sources, Internet of Things mechanisms, and smart meters, focus the need for a new system network. Consequently, considering decentralized energy reserves and transaction systems, Blockchain technology is regarded as a viable response to meet various Smart grid criteria. Meanwhile, several challenges should be considered as distributed ledger technology develops into a Smart grid. As a result, current Smart grid technologies, such as Internet of Things mechanisms and intelligent meters, would be used to facilitate Blockchain technology assimilation into Smart grids. On the other hand, the Blockchain Technology advantages and disadvantages in each area of Smart grids will be thoroughly examined. Furthermore, it is to certify that technological advances can impact the Smart grid performance. Blockchain technology used in Smart grid is divided into categories, as indicated in Fig. 2.

In the field of Information Technology adoption, numerous models have been proposed to study the behavior of end-customers towards various Information Technology products. These models include the Technology Acceptance Model by Davis, Diffusion of Innovation Theory by Rogers, and Technology Readiness Action by Parasuraman [5]. Recent studies in the Information Technology domain have focused on combining different theoretical models or factors that influence customer adoption behavior. This integration of models is supported in the literature to gain a more comprehensive understanding of customers' intent to adopt Information Technology applications. These models have been successful in determining Information Technology implementation and are widely discussed in the literature. However, most of these theories have been developed and tested in western countries, with limited research conducted in developing countries, including Pakistan.

The existing literature on Blockchain technology adoption is mostly conceptual expositions, and there is limited empirical research to extend the adoption theory and understand how the organization react to emerging technology. This paper stands out from others in terms of its contributions. It is a unique and early study that examines the impact of blockchain technology adoption on achieving better Energy performance using the Technology Organization Environment theory [6], as the underpinning framework. As there is limited information available in the literature on how blockchain technology has been adopted in the Smart Grid, the main drivers for achieving better energy performance through blockchain technology adoption remain unidentified. Therefore, this study aims to comprehensively understand the various decision-making factors influencing the Blockchain Technology adoption for the energy trading system in Pakistan and shed light on the two main research questions.

Research Question 01 What factors drive Pakistan energy firms to deploy Blockchain Technology in the Smart grid?

Research Question 02 Which factor (s) greatly relates to the user adoption intention in the Energy firm of Pakistan?

This study is unique as it presents the first empirical investigation into practitioners' intention to use Blockchain technology in the Smart grid-Pakistan context. This study aims to understand the decision-making factors that influence the intention to adopt blockchain technology for energy trading system in Pakistan. Research model based on the Technology Organization Environment framework is developed to examine the adoption intention of blockchain technology. One of the predicted consequences of this study is to assist scholars and practitioners in better understanding the key drivers of Blockchain Technology adoption in the Smart grid context. It leads to better insights in to how to move forward with technological development. The Technology Organization Environment framework has a solid hypothetical foundation, empirical support, and the ability to be applied to various Information System domains, even though precise components identified within the three dimensions may vary across diverse research. Due to its emphasis on technological, environmental, and organizational constructs that affect the decision to accept technological advancements, we selected the Technology Organization Environment in the Framework. Technology, environmental, and organizational factors affect whether a company adopts new technology. In the literature, several descriptive variables are commonly used to define organizational context, including company size, formalization, centralization, and difficulty of its managerial structure. The organizational enablers, such as the calibre of its individual resources and the quantity of inner slack assets, are critical to be investigated. The term "technological context" refers to inner and outside technologies pertinent to the company. This comprises the technologies already used within the company and the various technologies on the market. The environmental context is the setting in which a company conducts trade. It includes industry, competitors, entry to outside resources, and interactions with the government.

We argue that this study has a unique proposition as the primary empirical study for the practitioner's intention to use Blockchain Technology in the Smart Grid- Pakistan context. The Partial Least Square Structural Equation Modelling technique [7], was employed for the model validation and hypothesis inquiry. Partial Least Square is a soft Structural Equation Modelling technique that does not rely on any presumptions about data distribution. In real-world research initiatives, Smart Partial Least Square helps Structural Equation Modelling. Because it allows them to estimate composite models with multiple factors, indicator constructs, and structural routes without making distributional assumptions about the data, the PLS-SEM approach is well-liked by academics. However, Partial Least Square is a basic Structural Equation Modelling predictive technique that emphasizes prediction when estimating numerical models with structures intended to shed light on causal relationships. The present study also focuses on how this innovative technology could be effectively utilized. Insights for best practices would be the drivers of improvement. At this time, Blockchain Technology has progressed to the concept testing stage and is at the deployment stage. Initial adopters' insights could help to persist in the proof concept of digitization of production and distributions. Blockchain Technology can raise the efficiency of energy operations due to its traceability capabilities. Using Blockchain Technology in the energy sector improves safety, lowers prices for consumers and businesses, and fosters trust. Building digital rights management for Energy Management can be facilitated by a trust model in Blockchain Technology.

The paper structure of the remaining study is organized as follows: Section 2 describes the theoretical background, Section 3 discusses the proposed model and hypotheses development, Section 4 covers the research methodology, Section 5 clarifies the study findings, Section 6 discusses the major findings and implications of our study, while Section 7 concludes the study by addressing the study limitations and providing prospective research directions.

## 2 Theoretical background

### 2.1 Blockchain technology

Blockchain technology, conceived by Satoshi Nakamoto [8], has garnered immense attention for its remarkable features across various sectors. Its ability to securely record transactions between businesses has led to reduced transaction costs, enhanced supply chain transparency, and increased traceability in the manufacturing domain to combat counterfeiting. Energy firms can leverage Blockchain technology to create contracts embedded in digital codes, ensuring authentic data storage without the risk of deletion, revision, or tampering. Blockchain technology provides a higher level of validity, enabling digital signature verification and contract identification. The advantages of Blockchain technology extend to the energy sector, potentially eliminating the need for intermediaries. Peer-to-Peer transmission, persistency, automation, auditability, and immutability are among the benefits derived from the cryptographic hash nature of the distributed ledger, digital signature of smart contracts, and distributed network of consensus algorithms. The specific process depends on the consensus mechanism employed. Three phases of Blockchain technology applications can be distinguished: Blockchain 1.0, which involves the virtualization of digital currencies like Bitcoin; Blockchain 2.0, which incorporates smart contracts for transaction processes; and Blockchain 3.0, enabling a high degree of autonomy through decentralized autonomous organizations based on pre-defined complex rules.

Public/permissionless blockchains like Bitcoin and Ethereum allow anyone to participate and access ledgers, relying on proof of work (PoW) consensus mechanisms for data validation. In contrast, private/permissioned blockchains such as Hyperledger Fabric restrict data access to members, with proof of authority (PoA) consensus mechanisms where a single node generates new data blocks. Proof of stake (PoS) can also be used in private blockchains. Consortium blockchains strike a balance between public and private blockchains, allowing only verified members to validate blocks. Tendermint is widely used for seamless token exchange between different blockchains.

In terms of economic development, Blockchain technology can be divided into three main stages. The first stage, from 2009 to 2012, can be referred to as the "gestation period" when Bitcoin and the industrial ecology were formed. The second stage, from 2012 to 2015, marked the infancy of Blockchain technology as it gained public attention, and the system token bit was stripped away. The third stage, beginning in 2016, witnessed the exploration of industrial applications for Blockchain technology. Market research firm Gartner predicts that by 2020, the blockchain industry will reach $100 billion. Klas Schwab, the founder of the Davos Forum, optimistically forecasts that by 2025, 10 % of the world's total GDP will utilize blockchain storage techniques [9].

In conclusion, Blockchain technology offers decentralized solutions that can lower transaction costs, enhance security, increase transparency, and improve traditional systems in the energy sector. Its potential impact on energy firms is significant, and understanding the factors driving its adoption is crucial for the successful implementation of Blockchain technology in the Smart Grid context.

### 2.2 Peer-to-peer energy trading

A central entity handles the majority of traditional energy dealings. It will become increasingly complex as more prosumers admit to the energy markets. The trading between producers and customers will get more complicated as well. In most countries today, electricity is typically a monopoly industry. Electricity must be purchased through the electric power provider by all consumers. Because the electricity rate is fixed, users cannot choose the most cost-effective energy purchase plan on their own. The decentralized market now has a new opportunity, and blockchain technology can aid in developing a transparent and reliable electricity trading system. Customers and producers can connect directly for energy transactions on a distributed network. Introducing a novel distributed energy trading network based on Blockchain would alter power firms' roles in the energy markets. They would no longer be required to invest heavily in organizational management services for a bulky number of customers. Users would have different energy buying systems in a low-cost energy trading scenario. The use of Blockchain technology will alleviate issues that arise during the exchange process in the energy markets and provide additional benefits. It can, for example, increase trading transparency, improve the smooth operation of the power grid during point-to-point energy transactions, enhance demand feedback, billing and other operational processes, and preserve safety. Some blockchain-based peer-to-peer energy trading markets primarily employ blockchain to implement market auction mechanisms.

Based on the private blockchain, the study of E. Mengelkamp et al. [10], presented a Blockchain-based system network for users in local energy marketplaces. Local energy transfers never require the use of a central mediator. Their work focused on a 100-user proof of concept framework using Ethereum for a local transaction system for household photovoltaic power generation. The system regulates supply and demand using a market auction process. The market auction process is realized on its proposed market platform by deploying smart contracts, and payment is likewise completed on a blockchain network. Smart meters automatically measure and forecast each agent's demand and production capacity and then transmit them to each agent. Surplus demand is computed based on this information and transferred to the agent's blockchain account; however, members' data remains silent.

Hahn et al. [11], designed a Blockchain-based distributed power auction framework. This distributed auction system allows users to execute online auctions and achieve a dependable, protected, and translucent energy exchange. The network has two types of domains, vendor and buyer, and two tools: smart meter and smart contract. Smart contract automates the auction and payment procedures. While the smart meter monitors and checks the electricity flow during the transactions, ensuring that it is completed. Blockchain will record all information from sellers, bidders, and smart meters. When a vendor has extra open power that he desires to trade, he can start an auction and broadcast it on the blockchain. After receiving the new auction, purchasers could place a bid.

The study of Aitzhen et al. [12], focused on addressing security issues in distributed energy transactions on the smart grid by employing privacy protection methods. They proposed a private blockchain energy trading framework built on tokens instead of using an auction process to complete a transaction. The users could negotiate electricity costs without identifying their names using this method, and their personal information is adequately secured during the energy trading. The exchange system employs Blockchain technology, several signatures, and unidentified encrypted data flow to ensure anonymity. This trading system lacks a trusted third party, allowing participants to negotiate their prices. They are, however, permanently anonymous, and the information is encrypted to preserve their safety. Their study examined several safety and confidentiality provisions that must be met through a blockchain-based transaction system in-depth and the system's recommended countermeasures. This study could guide the creation of a blockchain-based transaction system.

Rather than using a market auction method, other Blockchain-based trading frameworks adjust energy pricing based on energy storage, trading, and current energy pricing.

Park et al. [13], presented a Blockchain-based Peer-to-Peer energy trading network that allowed users to trade electricity efficiently. The use of distributed ledgers to stock and validate energy labels was proposed in their study. Where energy is created and consumed will be identified by energy labels. The steps in the trading procedure are as follows. Foremost, Internet of Things devices would determine if members need to purchase/sell energy and automatically create a label. The label would then be emailed to all members of the transaction platform. When a trader chooses to trade with the label's creator, the label is confirmed, and the two members complete the transaction. Conclusively, the two parties' transaction records are created and added to a block that will be broadcast to the entire network. Depending on the trade conditions, the trading platform can compare and adjust the present marketplace pricing of power energy. It will also feature an algorithm to assist consumers/prosumers in determining the most cost-effective and high-quality energy alternative, allowing them to trade more successfully. The fundamental aim is to create an automated energy trading framework allowing consumers and prosumers to sell high-quality, low-cost energy anytime and anywhere.

Cheng et al. [14], proposed a Blockchain-based business framework that implements distributed pricing and peer-to-peer trading. They first designed a blockchain-based decentralized power market trading architecture, then focused on a pricing mechanism to enhance consumer supply and demand balance. Simultaneously, their study contrasts traditional power transaction techniques with Blockchain-based power techniques. On the other hand, the transparency and security of transaction information are essential benefits of Blockchain-based energy trading. Every node can access all the transaction archives, making the trade more transparent. Furthermore, information is stored on the blockchain, which is safer because all participating nodes synchronize it. Traditionally, transaction information is held by the central administration organization, and only the central authority has access to the transaction system. Conversely, a hostile attack on the central authority could reveal users' personal information and its malicious use, with disastrous consequences. The Blockchain applied in Peer-to-Peer trading is presented in Table 1.

**Table 1:** Blockchain applied In peer-to-peer trading.

Ref	Technology	Objective	Contribution
[15]	Hybrid Blockchain	Data Storage	Design a data storage system for energy internet based on a hybrid blockchain.
[16]	Blockchain Smart contract	Decentralized Grid Control	Accomplish the centralized design's control purpose while avoiding the problem; as the number of members increases, so does the cost and difficulty of controlling.
[17]	Blockchain	The Smart Grid Control	Blockchain records all information in the energy transaction procedure and powers smart meters as a control system.
[18]	Blockchain Smart meters Smart contracts	Consumer Data Protection	Implement a Blockchain technology on a smart grid system, which can protect consumer data.
[19]	Blockchain Smart Electricity meters	Data Protection	Improve data security in the energy system and prevent data from being misused maliciously.
[20]	Blockchain Smart meters Smart contracts	The Demand Side Grid Mgt	Use blockchain and smart contracts to keep a balance of power supply and demand.

### 2.3 Technology adoption models

Technological developments and progress have always played an essential role in a country's financial growth. Various studies have examined technology adoption models, focusing on customer adoption of renewable energy consumption through comparing perceived qualities and attitudes. Technological advancements in the energy sector can help the country's environment remain sustainable. The technology projects stated above are for improving society and customers' well-being, but they are contingent on technology acceptance. Because of its focus on technology, environment, and organization characteristics that affect the decision to accept technological advancements, the Technology Organization Environment Framework was established by Tornatzky, Fleischer, and Chakrabarti (1990) [6], and used in this study. Technology Organization Environment presents a more comprehensive picture of technology adoption. It incorporates human and non-human elements into a sole framework; the Technology Organization Environment framework outperforms traditional models such as the Technology Acceptance Model, Theory of Planned Behavior, and Unified Theory of Acceptance and Use of Technology model.

Fernando et al. [21], utilized the Technology Organization Environment model to investigate the drivers of Blockchain uptake and carbon performance. After passing the proof of concept stage, Blockchain draws early users who can benefit from it. Based on their significant findings, the primary impediments to industrial enterprises adopting DLT were a lack of senior management support and a lack of technological proficiency. Their results showed that enterprises did not attain low carbon performance due to a lack of competitive pressure to implement Blockchain. There was no evidence that early adopters of Blockchain were associated with low carbon performance. Their study's recommendations include taking the initiative to track energy consumption, participating in carbon credit transfers, and following carbon performance using Blockchain to increase business transparency and sustainability. A. Ahl et al. [22], obtained findings based on literature analysis and professional interviews, and an analytical model for Peer-to-Peer microgrids was established. They used the five-dimensional Framework: technological, economic, social, environmental, and institutional. Their study aimed to examine all the issues that Blockchain-based microgrids could face and the practical consequences for institutional development. Based on the significant findings, it is indicated that bridging the gap between technological and institutional readiness will necessitate the consideration of all measurements and interdependence. Gradual institutional transformation based on regulatory sandbox techniques is presented as a possible path to incorporate this multi-dimensionality, decreasing cross-sectoral silos and allowing interoperability between present and forthcoming systems. Their work adds to rising research in generating a more established institutional arch for Blockchain in the power sector by providing insight through holistic conceptualization.

To supplement these past studies on the Technology Organization Environment framework, our study aims to specify a comprehensive understanding of the several decision-making constructs that affect the adoption intention to use the Blockchain technology for the Smart Grid- Pakistan context. The recent literature on Technology adoption models is presented in Table 2.

**Table 2:** Technology adoption models.

Author	Model used	Major Findings
[23]	Technology Acceptance Model	Using Technology Acceptance Model with four variables (Perceived behavior, moral norms, awareness, and social norms), the study findings concluded the applicability of Technology Acceptance Model in the renewable energy sector in Iran. The findings indicate a significant positive correlation between the variables of intentions and a negative correlation with intentions in terms of social norms.
[24]	Technology Acceptance Model	The research work focused on examining the perceived usefulness of Blockchain Technology in energy transactions. The findings of the study revealed that users are strongly inclined towards the key aspects of security, ease of use, traceability, verifications, and digital transactions when utilizing Blockchain Technology. These findings have important managerial implications, highlighting the potential of Blockchain in shaping the future of energy transactions. The research underscores the significance of addressing users' concerns and preferences related to security, usability, and efficiency when implementing Blockchain technology in the energy sector.
[25]	Technology Acceptance Model	The study focused on exploring the potential of Blockchain Technology as a decentralized business model for energy firms, adopting a sharing economy perspective. The findings of the study highlighted that Blockchain technology enables decentralized and highly secure business transactions in the energy sector. By leveraging the features of Blockchain technology, such as transparency, immutability, and smart contracts, energy firms can establish trust, enhance efficiency, and streamline their operations. The research emphasizes the transformative role of Blockchain technology in reshaping traditional business models and facilitating a more decentralized and secure environment for energy transactions.
[26]	Extended Technology Acceptance Model	The individual constructs of the behavior model showed significant correlations with the intention to use Blockchain technology adoption, while their collective effect was found to be insignificant. The quality of the system and the perceived enjoyment exhibited a stronger correlation with the perceived usefulness construct.
[27]	Unified Theory of Acceptance and Use of Technology	The findings confirm that assurance correlates most strongly with the user's intention to accept Blockchain technology adoption. Additionally, effort expectancy and performance expectancy show significant correlations with all other technology and service quality constructs.
[28]	Theory of Planned Behavior, Diffusion of Innovations Theory	The findings confirm that service compatibility and perceived benefits play a significant role during Blockchain adoption. However, trialability and observability show insignificance during the adoption of blockchain transactions.
[29]	Diffusion of Innovations Theory	The study findings suggest that ventures would increase as the pioneer in blockchain appropriation, for example, oil trading will various provider layers. Using the rancher's case, the study predicted that a blockchain endorsement by one entity would apply standardizing pressure on various supply network elements.
Our Study	Technology Organization Environment	This study findings confirm that Cost saving, regulatory support and relative shows significant correlation for intention to use Blockchain in the Smart Grid. However, Traceability and Competitive Pressure were insignificant predictors.

## 3 Proposed model

The Technology Organization Environment framework has been developed to predict the adoption of Blockchain in the smart grid. The technological, organizational, and environmental dimensions are the three elements that are considered drivers of Blockchain adoption. The technical dimension includes all firm-related technologies, both already in use and available but not yet in use. Existing technologies are critical in the adoption procedure because they limit the scope and speed with which a corporation can modify its technology. Employee linkage structures, interfirm communication networks, and slack resources are all examples of organizational context. The environmental dimension is the industry in which a company conducts its business and the competitive pressure on the firm. The jurisdictive environment, as well as the industry structure and the existence or lack of technological service providers, all contribute to the environmental dimension. The industry's structure has been investigated in many ways. Rivalry, for instance, promotes the adoption of innovative technology. Furthermore, dominant value chain firms may impact the innovation of other value chain partners [30].

**Fig. 3:**
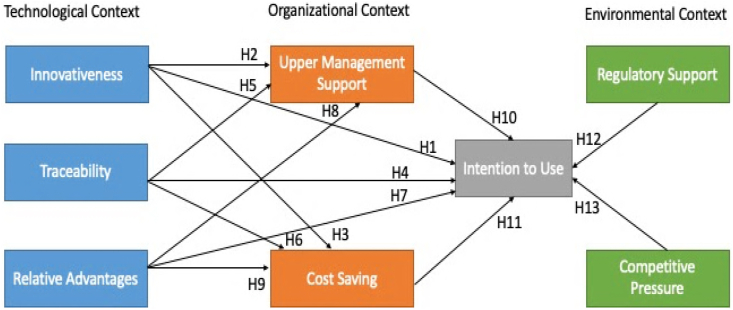
Research proposed model

The proposed model aims to utilize the Technology Organization Environment framework in the Energy Trading System of Pakistan to facilitate the acceptance of Blockchain Technology. By applying this framework, experts will gain a better understanding of the key drivers of Blockchain technology in Pakistan's energy sector and how to effectively progress with its development. The primary focus of the model is to explore successful utilization of this emerging technology. Valuable insights from early adopters will contribute to extending the proof of concept for digitization of manufacturing and distribution. Please refer to Fig. 3 for a visual representation of the proposed model.

### 3.1 Technological dimension

In the technological dimension, we consider innovativeness, traceability, and relative advantage as necessary for blockchain adoption in energy sector. The technology readiness index defines the innovativeness factor. It is a drive to be a technology visionary and leader [31]. Positive thinking can guide an expectant attitude toward innovation and a sense of assurance that it will provide efficiency and adaptability. The incentives of technology are used to quantify innovativeness. Energy trading and utilities will have a bright future but will take significant leaps. To save costs in the long run, the energy sector must be open to new ideas rather than being conceptually locked off by old ones. According to the previous findings, there is a favorable and considerable correlation between innovativeness and the desire to use technology [32]. Additionally, innovativeness shows a positive correlation with upper management support. As a result, there is a positive correlation between innovativeness and cost-saving [33]. Therefore, we postulate the following hypothesis.

H1 Innovativeness is positively associated with intention to use Blockchain technology.

H2 Innovativeness is positively associated with upper management support of Blockchain technology.

H3 Innovativeness is positively associated with cost-saving of Blockchain technology.

The application of Blockchain and other technologies, such as RFID and Internet of Things, can improve traceability, an essential quality aspect [34]. An electronic traceability framework that has gained prominence as a risk management instrument to maintain energy security, quality and supply chain integrity is credited with implementing integrated blockchain technologies. By tracking the chain of custody for grid materials, the Blockchain can increase efficiencies for utility companies. It may provide various services, including renewable energy origin certification and energy consumption tracking. Because a blockchain preserves every transaction history, the energy transaction can be traced back to its inception, preventing double-spending and lowering costs. The previous finding indicates a significant positive correlation between traceability and the intention to use Blockchain technology. Furthermore, traceability exhibits a positive correlation with upper management support [35,36]. As a result, there is a positive correlation between innovativeness and cost-saving [37]. So, we postulate the following hypothesis.

H4 Traceability is positively associated with intention to use Blockchain technology.

H5 Traceability is positively associated with upper management support of Blockchain technology.

H6 Traceability is positively associated with cost-saving of Blockchain technology.

A relative advantage is the extent by which an innovation is considered superior to its replaced concept. These characteristics are being utilized to clarify the end-user acceptability of technologies. We argue that the relative advantage is a significant determinant in the popularity of Blockchain technology for energy management. According to the literature, there is a positive correlation between customers' intentions to employ technology and perceived relative advantage. The immutable ledger can give safe and real-time information on energy usage. Traditional energy systems are slower than smart contract-based distributed ledger systems. The Blockchain can improve energy management system's and its transparency. As a result, Blockchain technology can give users more efficiency, lower costs, and more control over their energy source. According to our evaluation of past studies, there is a positive correlation between users receiving advanced relative aids and perceiving the technology to be of better utility. Moreover, relative advantage shows a significant correlation with upper management support [[38], [39], [40]]. As a result, there is a significant positive correlation between relative advantage and cost saving [41]. Therefore.

H7 Relative advantage is positively associated with intention to use Blockchain technology.

H8 Relative advantage is positively associated with upper management support of Blockchain technology

H9 Relative advantage is positively associated with cost-saving of Blockchain technology.

### 3.2 Organizational dimension

In the organizational dimension, we considered upper management support and cost saving as key constructs to accept Blockchain technology in the Smart Grid. As new regulatory requirements accompany blockchain adoption, top management support is critical. The upper management support plays a vital role in advising on the appropriateness of technology deployment to boost the overall performance of the energy trade. The previous findings indicate that upper management support favors the intention of using technology. Technology costs have eternally been a significant factor in its adoption. Emotive efforts, time, and capital employed by self-service technologies are all cost savings. It is "the degree to which a user believes that a specific method would reduce money spent on running the service". According to M. L. Meuter et al. [42], one of the sub-categories that drive client self-service choice is saved monetary items. In previous studies, electronic commerce and self-service technologies have been shown to lower transaction costs. Acceptance of innovative Blockchain technology can reduce transaction expenses such as security (for example, data encryption) and distribution expenses (for example, e-logistics service). Accordingly, there is a positive correlation between cost savings and the intention to use technology [43]. Thus, we assume the following hypotheses.

H10 Upper management support is positively associated with intention to use Blockchain technology.

H11 Cost saving is positively associated with intention to use Blockchain technology.

### 3.3 Environmental dimension

In the environmental dimension, we consider regulatory support and competitive pressure as an important construct for blockchain adoption in the energy sector. According to Guo et al. [44], challenges relating to regulation and implementing decentralized systems remain unaddressed, and industry standards must be developed quickly. In recent studies on blockchain adoption, the regulatory environment was one of the essential indicators. Competitive pressure refers to a firm's perception of pressure from competitors to accept novice technology, and it has also been found to be a crucial driver of technological dissemination. Corporations are prepared to adopt new technologies if their competitors have previously done so regarding industrial behavior. They recognize that technology may assist them in becoming more cost-effective to get a competitive advantage [45]. Hence, the following hypotheses are formulated.

H12 Regulatory support is positively associated with intention to use Blockchain technology.

H13 Competitive pressure is positively associated with intention to use Blockchain technology.

## 4 Research methodology

**Fig. 4:**
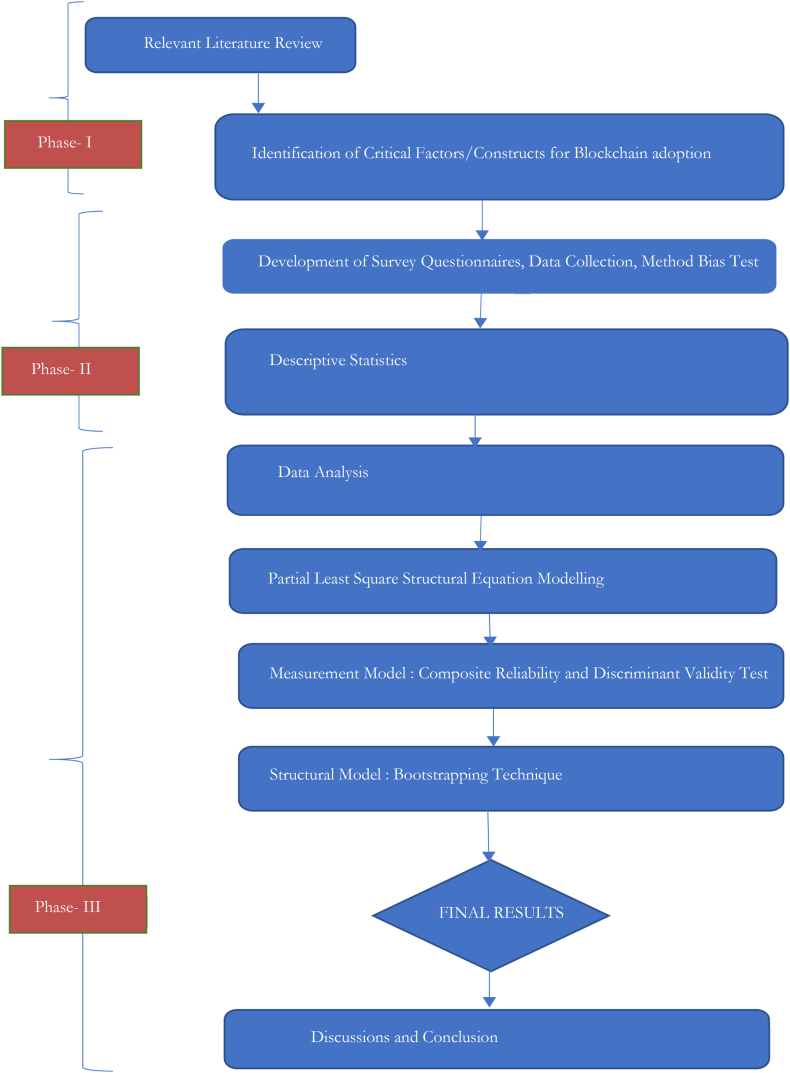
Flow chart of research methodology

The research methodology employed in this study followed a systematic approach based on the Technology Organization Framework to investigate the impact of blockchain-powered grids on achieving sustainability and efficiency. The methodology consisted of several key steps. Based on the literature review, critical constructs were identified and formed the basis for developing survey questionnaires. Descriptive statistics were employed to analyze the collected data, providing insights into the relationships between the identified constructs. To further analyze the data, SmartPLS was utilized, encompassing both the measurement model and the structural model. The results obtained from the analysis were then interpreted, leading to a comprehensive discussion of the findings. Finally, the study concluded with a comprehensive summary of the results, implications, and recommendations for future research. The flowchart illustrating the research methodology is provided in Fig. 4.

### 4.1 Instrument development

The proposed model in this study is made up of three-dimensional multi-item constructs. All of the items were taken from previous literature. A survey method was used for the data collection. Then, the relationships have been tested between factors in the proposed model. All scales, ranging from 01 to 05, were evaluated using the 5-point Likert rule. First, we contacted five (05) professionals as a pretest to validate the instruments before conducting the survey. Based on the input, we made minor changes to improve the operation of the times in the questionnaire. The construct details are presented in Table 3.

**Table 3:** Construct measurements.

Construct	Code	Question	Ref
Innovativeness	INN1	You keep up with the modern technological developments in your areas of interest, such as blockchain.	[46]
	INN2	You find you have fewer problems than other people in making BCT work for you.	
Traceability	Trace1	Blockchain can potentially improve utility providers' efficiencies by tracking the chain of custody for grid materials.	[47,48]
	Trace2	Blockchain offers traceability of energy produced and consumed at each endpoint, informing consumers about the origins and making energy charges more transparent.	
	Trace3	Blockchain solutions can provide several services such as origin certification and traceability for energy consumption.	
Relative Advantage	Adv1	Blockchain can provide safe and real-time updates of energy usage data.	[49,50]
	Adv2	The smart contract-based Blockchain-based DLT system is faster than the traditional energy system.	
	Adv3	BCT can provide customers with higher efficiency and control over their energy source	
Upper Management Support	Mgt1	Upper management encourages blockchain technology adoption in the energy sector.	[51]
	Mgt2	Upper managers are willing to take risks for the acceptance of blockchain technology.	
	Mgt3	Upper managers actively respond and pay attention to new blockchain projects.	
Cost Saving	Cost1	A digital ledger makes it possible for the energy sector to reduce costs while improving reliability and distribution efficiency.	[52,53]
	Cost2	Blockchain can create a stable energy market with lesser electricity costs by connecting customers directly to the grid.	
	Cost3	Blockchain will reduce transaction cost in the energy sector.	
	Cost4	Distributed ledgers are cost-effective.	
Regulatory Support	Reg1	The relevant authorities have to support blockchain adoption in the energy sector.	[54,55]
	Reg2	The Government introduced relevant policies to boost Blockchain development.	
	Reg3	There is lawful support for the use of BCT in the energy sector.	
	Reg4	The present regulations are enough to protect the use of BCT in the energy sector.	
Competitive Pressure	Comp1	Blockchain's exciting features and competitive pressures push the energy sector to look into its adoption.	[56]
	Comp2	The energy sector believes that using BCT applications to gain competitiveness is significant when making strategic decisions.	
	Comp3	The advent of Blockchain in developed countries is emerging as a new instrument to better the traditional energy system.	
Intention to use	Int1	The energy sector will use blockchain technology	[57]
	Int2	It is expected that your energy sector would benefit from Blockchain applications in the service and manufacturing processes.	
	Int3	By adopting Blockchain, the energy sector would provide better service.	

### 4.2 Data collection

In May 2021, the survey questionnaires were physically distributed to the 17 Peshawar Electric Supply Chain Company (PESCO) departments. This study does not convert the written questionnaire into our native language Urdu. The Employees working in Pakistan's Energy Sector use English as the communication and official language. Overall, we received 186 survey questionnaires from the experts and found that the usual completion time is around 05 min, comparable to the assessed end period based on the pilot test. We did not consider the partial and short-term response in our study. Consequently, we didn't take the 18 responses into account because they were incomplete. Furthermore, we checked the remaining replies and discovered that no two replies to the same item had the same score. Finally, we considered 168 valid responses for further research. As the Blockchain is a novice technology for Pakistan. Table 3 shows that the largest job location group was Management, accounting for 54 out of 168 respondents. Their feedback can help us in the managerial implications of our study. It was found that the sample of 168 respondents met the least criteria of five observations per parameter. For the structural equation modelling study, we used 25 parameters and a minimum sample size of 165 respondents [7]. The respondent's profile details are presented in Table 4.

**Table 4:** Respondent profile.

Demographic Variable	Category	Frequency	Percentage (%)
Gender	Male	126	75.0
	Female	36	21.4
	Prefer not to say	6	3.6
Age (years)	18–23	50	29.8
	24–28	41	24.4
	29–34	50	29.8
	35–40	17	10.1
	Above 40	10	6.0
Experience (years)	Less than 1 year	23	13.7
	1–4 year	32	19.0
	5–7 year	46	27.4
	8–10 years	41	24.4
	Beyond 10 years	26	15.5
Job Location	Grid Station Operation	16	9.5
	Information Technology	20	11.9
	Management	54	32.1
	Operations	47	28.0
	Others (Audit Division, Finance Directorate, Planning & Engineering, Project Construction etc.)	31	18.5

### 4.3 Common method bias

Harman's single-factor test was applied to investigate the possibility of common method bias. The investigation findings indicated that 42.816 percent of the data difference was captured through the primary factor. As a result, the outcome is less than 50 %. There was no common technique bias issue, as one might assume [58]. Moreover, we applied a VIF full collinearity test to check multicollinearity. The findings also indicate that the factor's highest VIF was less than the threshold of 5. The structural model can also be used to induce endogeneity. As a result, we executed a Ramsey regression equation error test and confirmed that there was no endogeneity problem [59]. The proposed model VIF values are presented in Table 5.

**Table 5:** VIF values.

Inner VIF	Comp	Cost	INN	Int	Reg	Adv	Trace	Mgt
Comp				1.756				
Cost				2.467				
INN		2.329		2.531				2.329
Int								
Reg				1.568				
Adv		1.759		2.935				1.759
Trace		2.316		2.705				2.316
Mgt				3.243				

### 4.4 Descriptive statistics

The descriptive statistics provided in Table 6 offer valuable insights into the distribution and characteristics of the variables under study. The variables have a scale from 1 to 5, with mean values ranging from 2.131 to 3.446. The median values are mostly around 3, indicating a central tendency towards the middle of the scale. The standard deviation values range from 0.923 to 1.283, suggesting variability in the responses within each variable. The excess kurtosis values are negative for all variables, indicating flatter distributions with lighter tails compared to a normal distribution. This suggests that the data may have fewer extreme values or outliers. The skewness values are mostly negative or close to zero, indicating a slight left skew or symmetry in the distributions. This suggests that the majority of the data is concentrated towards the left side of the distribution. Overall, the provided descriptive statistics seem reasonable and provide insights into the distribution and characteristics of the variables.

**Table 6:** Descriptive statistics.

Code	Scale Min	Scale Max	Mean	Median	Standard deviation	Excess kurtosis	Skewness
Adv1	1	5	3.101	3	1.168	−1.086	0.027
Adv2	1	5	3.173	4	1.282	−1.224	−0.191
Adv3	1	5	3.155	3	1.268	−1.144	−0.082
Comp1	1	5	2.548	2	1.174	−0.994	0.23
Comp2	1	5	2.351	2	1.124	−0.606	0.542
Comp3	1	5	2.506	2	1.018	−0.565	0.257
Cost1	1	5	3.286	3	1.113	−0.886	−0.063
Cost2	1	5	3.345	3	1.134	−0.861	−0.167
Cost3	1	5	3.238	3	1.211	−1.011	−0.062
Cost4	1	5	3.327	3	1.157	−0.701	−0.224
Int1	1	5	3.179	3	1.212	−1.043	−0.105
Int2	1	5	3.321	3	1.177	−1.042	−0.096
Int3	1	5	3.246	3	1.283	−1.237	−0.074
Mgt1	1	5	3.399	4	1.254	−1.208	−0.188
Mgt2	1	5	3.095	3	1.264	−1.081	−0.02
Mgt3	1	5	3.196	3	1.283	−1.138	−0.1
Reg1	1	5	2.28	2	1.011	−0.171	0.598
Reg2	1	5	2.315	2	1.092	−0.104	0.702
Reg3	1	5	2.131	2	0.923	0.11	0.652
Reg4	1	5	2.387	2	1.165	−0.688	0.459
Trace1	1	5	3.161	3	1.146	−0.796	−0.152
Trace2	1	5	3.387	4	1.159	−0.602	−0.404
Trace3	1	5	3.446	4	1.106	−0.895	−0.209
Inn1	1	5	3.113	3	1.141	−0.771	−0.176
Inn2	1	5	3.137	3	1.185	−0.971	−0.008

## 5 Results

For the current analysis, the Partial Least Square Structural Equation Modelling was used. It is beneficial for structural equation modelling in practical research projects. The partial least square structural equation modelling approach is popular among academics because it permits them to estimate composite models with several factors, indicator constructs, and structural paths without making distributional suppositions about the data. On the other hand, partial least square structural equation modelling is a fundamental predictive method that highlights prediction when estimating numerical models with structures meant to provide causal clarifications.

### 5.1 Reliability and validity test

**Fig. 5:**
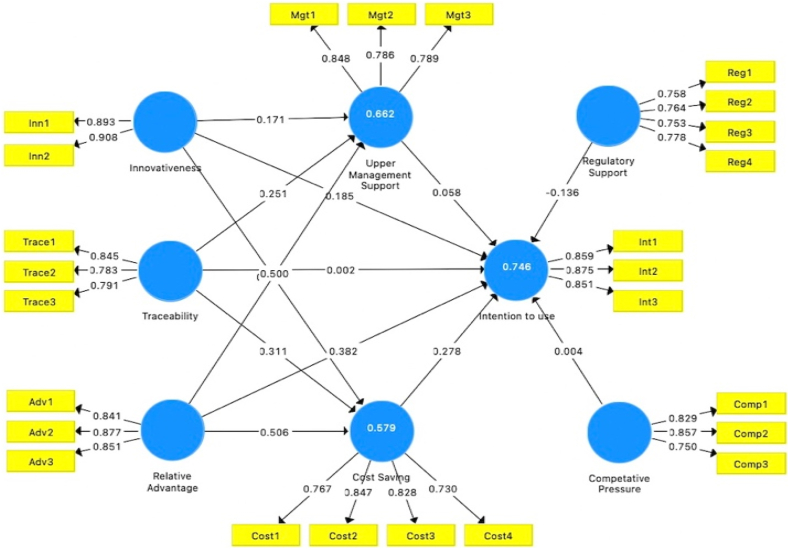
Measurement model

This study assessed all constructs using the measurement model validity recommendations. First, we determined the significance level of the loading factor; the criterion for every item loading is 0.70 or higher. As shown in Fig. 5, the findings reveal that all values meet the standard. After the factor loading test, all constructs were subjected to the composite reliability and average variance extracted tests. The study findings show that all values meet the minimum thresholds of 0.70 for Composite reliability and 0.50 for Average Variance Extracted [60], as presented in Table 7. Discriminate validity was examined to investigate how measurement elements in a conceptual model differed. The results show substantial validity and could be used to assess structural model measurements. Table 8 presents the discriminate validity.

**Table 7:** Factors reliability and validity.

Factors	Alpha	Rho_A	Composite Reliability	Average Variance Extracted
Competitive Pressure	0.744	0.745	0.854	0.662
Cost Saving	0.804	0.809	0.872	0.631
Innovativeness	0.767	0.770	0.896	0.811
Intention to use	0.827	0.827	0.896	0.742
Regulatory Support	0.763	0.766	0.848	0.583
Relative Advantage	0.819	0.821	0.892	0.734
Traceability	0.732	0.736	0.848	0.651
Upper Management Support	0.735	0.742	0.850	0.653

**Table 8:** Discriminant validity.

Fornell-Larcker Criterion								
								
Competitive Pressure	0.814							
Cost Saving	−0.418	0.794						
Innovativeness	−0.483	0.567	0.901					
Intention to use	−0.488	0.748	0.671	0.862				
Regulatory Support	0.555	−0.388	−0.423	−0.509	0.763			
Relative Advantage	−0.465	0.715	0.611	0.795	−0.426	0.857		
Traceability	−0.424	0.642	0.724	0.628	−0.320	0.609	0.807	
Upper Management Support	−0.545	0.680	0.658	0.717	−0.439	0.757	0.679	0.808

### 5.2 Structural model

**Fig. 6:**
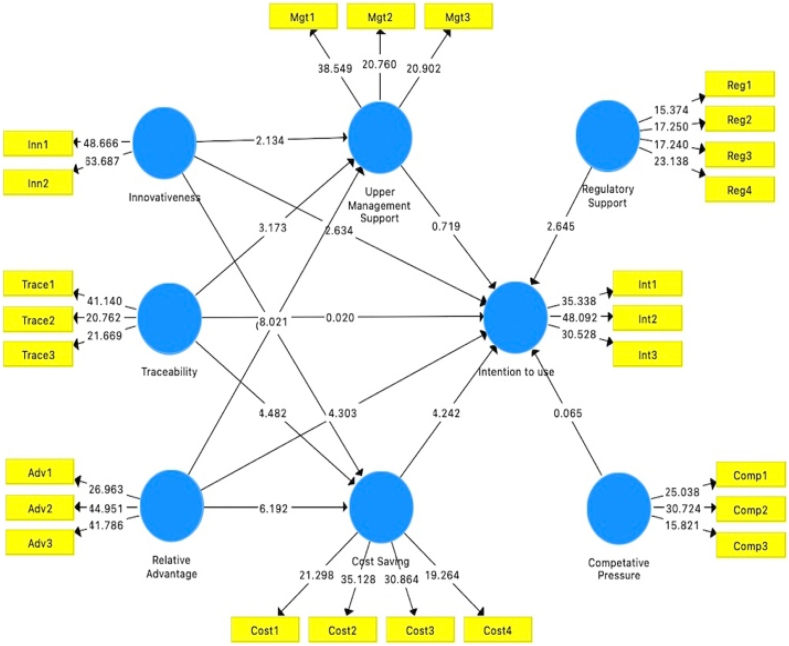
Structural model

In the following stage of analyzing the Structural Equation Modelling, the bootstrapping test was applied to test the significance of the route coefficients. Subsamples (5000) were evaluated with replacements to avoid errors in the bootstrapping technique, providing approximate t-values for significance testing of the conceptual model. The bootstrapping approach for structural equation modelling approximates data normality, as shown in Fig. 6. Latent variables predict 74.6 percent of the variation in intention to use. The final hypothesis development decision is presented in Table 9. PLS predict, a holdout sample-based technique for generating case-level prediction on an item using the PLS Predict with a 10-fold process to check for predictive capacities, was presented by Hair et al. [60]. According to Table 10, the PLS model's majority of errors were lower than the LM model's, representing that our proposed model has strong predictive power.

**Table 9:** Hypotheses results.

	Original Sample	Mean	STDEV	T Statistics	P Values	Decision
Innovativeness - > Intention to use	0.185	0.180	0.073	2.539	0.011	Accepted
Innovativeness - > Upper _Mgt Support	0.171	0.173	0.075	2.284	0.023	Accepted
Innovativeness - > Cost Saving	0.032	0.030	0.078	0.414	0.679	Rejected
Traceability - > Intention to use	0.002	0.010	0.087	0.019	0.985	Rejected
Traceability - > Upper Mgt Support	0.251	0.252	0.084	2.981	0.003	Accepted
Traceability - > Cost Saving	0.311	0.314	0.071	4.375	0.000	Accepted
Relative advantage - > Intention to use	0.382	0.386	0.084	4.536	0.000	Accepted
Relative Advantage - > Upper Mgt Support	0.500	0.501	0.059	8.466	0.000	Accepted
Relative advantage - > Cost Saving	0.506	0.506	0.074	6.823	0.000	Accepted
Upper Mgt Support - > Intention to use	0.058	0.051	0.074	0.790	0.430	Rejected
Cost Saving - > Intention to use	0.278	0.274	0.071	3.936	0.000	Accepted
Regulatory support - > Intention to use	−0.136	−0.138	0.048	2.804	0.005	Accepted
Competitive Pressure - > Intention to use	0.004	0.002	0.059	0.062	0.951	Rejected

**Table 10:** PLS-predict.

	PLS		LM		PLS - LM		
	RMSE	MAE	RMSE	MAE	RMSE	MAE	Q^2^_predict
INT1	0.827	0.630	0.864	0.653	−0.037	−0.023	0.540
INT2	0.851	0.678	0.874	0.701	−0.023	−0.023	0.484
INT3	0.921	0.712	0.988	0.764	−0.067	−0.052	0.488

### 5.3 Structural model assessment

According to the study findings in Table 9, the result of innovativeness on the intention to adopt DLT was (β = 0.185, T = 2.539, P = 0.011). So, H1 is supported. According to the findings, there was a positive correlation between innovativeness and upper management support (β = 0.171, T = 2.284, P = 0.023), indicating that H2 is supported. Subsequently, the outcome between innovativeness and a cost saving was (β = 0.032, T = 0.414, P = 0.679). Thus, H3 is not supported. The next outcome of traceability on intention to use Blockchain Technology was (β = 0.002, T = 0.019, P = 0.985). Thus, H4 is not supported. The outcome between traceability and upper management support was (β = 0.251, T = 2.981, P = 0.003). So, H5 is supported. Subsequently, the outcome between traceability and cost saving was (β = 0.311, T = 4.375, P = 0.000). Accordingly, H6 is supported. The next outcome of relative advantage on intention to use Blockchain technology was (β = 0.382, T = 4.536, P = 0.000). Therefore, H7 is supported. The outcome between relative advantage and upper management support was (β = 0.500, T = 8.466, P = 0.000). So, H8 is supported. The outcome between relative advantage and cost saving was (β = 0.506, T = 6.823, P = 0.000). Accordingly, H9 is supported. The next outcome of upper management support on intention to use Blockchain technology was (β = 0.058, T = 0.790, P = 0.430). Therefore, H10 is not supported. The outcome of cost saving on intention to use Blockchain technology was (β = 0.278, T = 3.936, P = 0.000). Hence, H11 is supported. Similarly, the outcome of regulatory support on intention to use Blockchain was (β = −0.136, T = 2.804, P = 0.005). Therefore, H12 is supported by the study. Finally, the outcome of competitive pressure on intention to use Blockchain technology was (β = 0.004, T = 0.062, P = 0.951). Thus, H13 is not supported by the study.

## 6 Discussion

### 6.1 Major findings

The study findings confirmed that innovativeness is positively associated with intention to use Blockchain technology and supported by the study of [61]. Our study findings further confirm the positive association between innovativeness and upper management support of Blockchain technology [51]. On the contrary, there was no significant association found between innovativeness and cost-saving in the context of blockchain technology. The study findings suggest that the adoption intention of Blockchain in the Energy Sector of Pakistan is still in its early stages, indicating its nascent state. The subsequent traceability analysis revealed an inconclusive correlation with the intention to use, deviating from the findings of other studies [62,63]. The study findings confirmed that traceability is indeed positively associated with upper management support of blockchain technology [64]. The study findings further validate that traceability is positively associated with cost-saving in the context of blockchain technology. Based on the study results, it can be suggested that Blockchain technology can aids in tracing real time energy transactions [65]. In a blockchain-based Smart grid, transactions can be recorded on a transparent ledger accessible to regulators and other energy firms. Our study findings confirm a positive association between relative advantage and the intention to use Blockchain technology [51]. There was no significant association found between the next upper management support and the intention to use Blockchain for the Smart grid [66]. So, marketing agencies should focus on awareness about the revolutionary Blockchain technology and its potential energy usage applications in the Energy Sector of Pakistan. Our study findings, in line with the support of another study [62], provide evidence of a positive correlation between cost saving and the intention to use Blockchain technology. Therefore, it can be suggested that Blockchain technology adoption in the Smart grid can reduce costs for energy firms. Our study findings confirm a positive association between regulatory support and the intention to use Blockchain technology. It is indicated that the relevant authorities support the most promising technology in the energy sector. The present policies are adequate to safeguard the usage of Blockchain in the energy sector of Pakistan. Our study findings confirm that competitive pressure has no significant association with the intention to use Blockchain technology, which deviates from the findings of the other study [51].

Based on our study findings, it can be suggested that the adoption of Blockchain in Smart grids is particularly suitable for developing economies, as it presents opportunities for increased efficiency, transparency, and decentralized energy management. The Blockchain sits amongst a cluster of fast-moving disruptive technologies that are said to comprise the 4th industrial revolution firms. Consequently, it can be stated that the Blockchain is getting attention as a promising technology that has the ability to revolutionize the future of the energy sector and has emerged as a novel marketplace pattern.

### 6.2 Theoretical implications

The emerging digitalization adoption in the energy sector that motivated by Ying et al. [67], is the impetus for our study. They claimed that an urgent need existed for empirical research to enhance the current status of Blockchain research, which is mostly exploratory. In fact, the majority of the Blockchain literature to date has taken the form of a literature review (Hughes et al. [68], and Min et al. [69], are two examples) or is conceptual (Francisco et al. [70], is another example). Even though some researchers have made a greater effort to gather empirical data, many investigations are rather constrained and concentrate on a single subject, such as those by Ying et al. [67], qualitative research like those by Wang et al. [71], or Unified Theory of Acceptance and Use of Technology frameworks like those by Queiroz et al. [72]. This study contributes to the existing body of knowledge by providing insights into the understanding of Blockchain adoption in the context of Smart Grids. With the theoretical lens of the Technology Organization Environment framework and empirical data from the Pakistan Energy Sector, have added diversity to the literature on adoption models for technological advancements using an empirical method. In the present study, we proposed a model to understand better how Blockchain technology is being adopted in Energy Management. Our proposed model was based on the Technology Organization Environment framework to fill a research gap in Blockchain technology acceptance for Smart grids. The primary contribution of this research study is to establish a solid groundwork for future research on Blockchain technology. Furthermore, it is important to highlight that this research study aims to contribute to the limited empirical research and literature in the field of Blockchain technology. While there are a few studies available for comparison that focus on Blockchain technology adoption in general, it is worth noting that they were not specifically conducted within the energy sector. Based on the study findings, the implementation of Blockchain technology in the Smart grid holds the potential for cost-effectiveness, regulatory compliance, improved energy process efficiency, and enhanced trust. Furthermore, the use of a trust model in Blockchain technology can facilitate the development of digital rights management for Energy Management. Developers and professionals can benefit from cost reduction and increased trust when adopting this revolutionary technology. Researchers can further expand on this research by extending our conceptual model to encompass a diverse range of cross-cultural nations.

### 6.3 Managerial implications

The study findings indicate that the proposed model has a strong descriptive impact, with significant results (R^2^ = 0.746 and R^2^ adjusted = 0.735), explaining a substantial 74.6 percent variation in the intention to use Blockchain technology. Additionally, the study reveals a significant impact on cost-savings, with a variance of (R2 = 0.579 and R^2^ adjusted = 0.572). Similarly, Upper Management Support also demonstrates a notable variance, with results of (R^2^ = 0.662 and R^2^ adjusted = 0.656). It is noteworthy that emerging economies have started exploring the adoption of Blockchain technology within their organizations. The study findings indicate a significant positive correlation between innovativeness and the intention to use Blockchain technology in the Smart grid. This highlights the importance of raising awareness about Blockchain technology adoption in developing countries such as Pakistan. The traceability and relative advantage of Blockchain are correlated with cost savings and upper management support, making it a valuable tool for tracing energy transactions. A blockchain-based Smart grid allows transactions to be recorded on an open ledger that regulators and other energy firms can view. However, the study shows that upper management support is not significantly correlated with the intention to use Blockchain technology for the Smart grid. Hence, marketing agencies should prioritize raising awareness about the transformative capabilities of Blockchain technology and its various applications in the energy sector, particularly in emerging economies. The intention to use Blockchain technology in the Smart grid is significantly correlated with cost-saving benefits and regulator support, indicating that its adoption can lead to cost reductions for energy firms. Moreover, the study indicates that it is essential for relevant authorities to actively endorse and support the most promising technological advancements in the energy sector. Although the current strategies effectively ensure the secure utilization of Blockchain technology in the energy sector, the adoption of Blockchain in the Smart grid shows no significant correlation with competitive pressure. This lack of correlation may explain the slow adoption process of Blockchain technology in Pakistan. In conclusion, Blockchain technology offers a smart and secure method to record and verify transactions without the need for a central authority. It improves safety, data transparency, and cost efficiencies in a firm's network. With Blockchain technology and Internet of Things devices, customers can directly buy and sell energy from the grid, bypassing traditional vendors.

## 7 Conclusion and future work

Taking a holistic perspective, this study provides an overview of the key constructs to consider within the Technology Organization Environment paradigm. In response to Research Question 01, the findings confirm a significant positive correlation between innovativeness, relative advantage, cost saving, and regulatory support with the intention to use Blockchain Technology in the Smart grid. Furthermore, the study reveals that innovativeness, traceability, and relative advantage notably correlate with Upper Management Support. Consequently, there is a positive correlation between traceability and relative advantage with Cost Savings. Regarding Research Question 02, the study concludes that Relative advantage plays a crucial role in the acceptance of Blockchain Technology for energy management. These findings highlight the importance of considering these correlations when implementing Blockchain Technology in the energy sector. As a result, both researchers and experts will find this a helpful study. As future work, this study examines a few variables inside the Technology Organization Environment model; however, an extension of the Technology Organization Environment could potentially contribute to the findings. The limitation of this study is that the survey questionnaires were only physically distributed to the 17 departments of Peshawar Electric Supply Chain Company. As a result, the data collected in this study solely represents the Peshawar region, where there is a lower representation of women in the workforce. To improve the generalizability of the findings, future research could include other major cities such as Karachi and Lahore. As a research protocol, we suggest developing case studies and field data such as surveys. Our research model demonstrate the significance of conducting further research on Blockchain technology and expanding its application to a broader range of countries worldwide. Our findings showed that Blockchain Technology acceptance by energy management professionals is still in its initial stages. Future research should look into the connection between Blockchain Technology awareness and the adoption of Blockchain Technology applications in the energy sector, among other things. Each tier of users in the energy industry may adopt the new Blockchain Technology differently. More research is needed into the energy industry's connectivity, particularly in developing a credit risk model. According to reports, Blockchain Technology eliminates inter-organizational intermediaries and establishes trust through networked nodes. More research is needed to determine the effect of data safety, integrity, and confidentiality on acceptance decisions and Blockchain Technology role in preserving sensitive information. In addition, the fundamental qualities of Blockchain Technology might need to be evaluated in terms of feasibility for acceptance from multiple viewpoints, such as interoperability and pricing. As a result, technology will drastically alter activities, and the energy sector must be prepared. There is remarkable scope for further study on Blockchain Technology adoption. The present study is conducted only for the energy trading system. We may take other technologically advanced nations in the future, like a cross-sectional comparative study of Pakistan with the United State of America and United Kingdom. The results of such a study will be more enjoyable. We argued that little research had been conducted on the cost of Blockchain Technology adoption apart from prototype research.

In the future, further study is required on the same technology; companies planning to incorporate Blockchain Technology into their traditional business would need more attention. In the future, we may incorporate Blockchain Technology with other technologies like big data and Artificial Intelligence to better understand the effect of privacy, the trustworthiness of data, and safety, as well as Blockchain Technology's responsibility in protecting sensitive data for organizational management. The results of such studies will be more helpful for energy firms. The current study is based on the Technology Organization Environment Framework. In the future, we can integrate other essential constructs such as Information System success theory, Performance Expectancy theory, diffusion of innovation theory, etc. The result of such important theories in this area for the energy trading system will be more substantial. The adoption of Blockchain Technology in the smart grid context has gained momentum. We argue that energy consumption and transaction transparency could be stored and tracked for those who have accessed the nodes. For example, energy firms can utilize Blockchain technology to monitor power flows from upstream and downstream energy supply chains with the support of an electrical network. Our study has proposed Blockchain energy adoption in the smart grid that investigated the enablers and performance from an organizational context. We do not utilize the experimental design to examine the adoption of Blockchain Technology in the innovative grid platform. It is suggested that future studies need to include technical variables on the specific power distribution network and resources, including coefficients estimation, electricity tariff and practical cost saving of electricity bills before and after technological adoption. Furthermore, it is critical to observe the flexibility and reliability of the electrical network to generate and distribute the energy to the consumers. Blockchain adoption has benefited energy stakeholders by facilitating peer-to-peer energy and electricity trading using smart contracts in real-time.

## Data availability statement

Data will be made available on request to corresponding authors.

## Funding

This work was funded by the Researchers Supporting Project Number (RSPD2024R564) 10.13039/501100004831King Saud University, Riyadh, Saudi Arabia.

## Institutional review board statement

Not applicable.

## Informed consent statement

Not applicable.

## CRediT authorship contribution statement

**Nazir Ullah:** Writing – original draft, Visualization, Validation, Resources, Formal analysis, Data curation, Conceptualization. **Waleed Mugahed Al-Rahmi:** Writing – review & editing, Writing – original draft, Visualization, Validation, Supervision, Software, Resources, Methodology, Investigation, Funding acquisition. **Fahad Alblehai:** Writing – review & editing, Visualization, Validation, Supervision, Methodology, Investigation. **Yudi Fernando:** Writing – original draft, Supervision, Investigation, Conceptualization. **Zahyah H. Alharbi:** Visualization, Validation, Software, Data curation, Conceptualization. **Rinat Zhanbayev:** Writing – review & editing, Resources, Methodology, Formal analysis. **Ahmad Samed Al-Adwan:** Validation, Resources, Conceptualization. **Mohammed Habes:** Project administration, Methodology, Investigation.

## Declaration of competing interest

The authors declare that they have no known competing financial interests or personal relationships that could have appeared to influence the work reported in this paper.
