# Supplementary material for: Blockchain-powered grids: Paving the way for a sustainable and efficient future
Source: Heliyon. 2024 May 21;10(10):e31592. doi: 10.1016/j.heliyon.2024.e31592 (PMC11145481; doi:10.1016/j.heliyon.2024.e31592)
Supplement: Multimedia component 1 [file mmc1.docx]

**Appendix (Questionnaire TOE Model)**

1. Blockchain technology is easy to use
2. You think blockchain technology is faultless
3. It is easy to do multitask through blockchain quickly
4. Blockchain is easy to use the than traditional Energy management system
5. Blockchain can help firms for fast transactions
6. Blockchain can bring transparency in firms
7. Blockchain can help in anti-counter measures
8. Blockchain can help you to reach stock in a real time
9. In your opinion, blockchain is necessary for Energy sector of Pakistan
10. You think, blockchain will improve the traditional energy management system
11. Distributed ledger will reduce transactions cost in the firms
12. Distributed ledgers are cost-effective
13. Distributed ledgers are compatible for improving supply chain efficiencies and cost saving
14. Blockchain ledgers are compatible with most aspects of Energy management firms
15. Other people come to you for advice to use blockchain technology for access at your firm.
16. You will use blockchain to access your firm database without any help
17. Firms will use distributed ledgers very well
18. It is expected that Pakistan Energy firms will take advantages from the blockchain application in the manufacturing and service operations.
19. By developing blockchain technology, Energy sector will increase resource usage and provide better services
20. Please tell me about your Designation, Experience, Area and C
